# An ergonomic study of arborist work activities

**DOI:** 10.1016/j.heliyon.2024.e26264

**Published:** 2024-02-15

**Authors:** Eva Abramuszkinová Pavlíková, William Robb, Jakub Šácha

**Affiliations:** a Department of Engineering, Faculty of Forestry and Wood Technology, Mendel University in Brno, Zemědělská 3, 613 00, Brno, Czech Republic; b Department of Statistics and Operational Analysis, Faculty of Business and Economics, Mendel University in Brno, Zemědělská 3, 613 00, Brno, Czech Republic

**Keywords:** Arboriculture, Physical and cognitive workload, Work measurement, Grip strength, Spatial awareness, Work safety

## Abstract

Arborists work in high-risk environments, particularly when climbing trees, where a combination of grip strength and resistance to psychological stress are important attributes for safety. This study investigated the physical and cognitive activities of arborists combined with selected workload factors such as blood pressure, pulse, handgrip strength, and other anthropometric measurements, including manual dexterity and spatial awareness. The sample included 10 participants aged 17–48 years. Blood pressure was negatively correlated with handgrip strength after the activity had been performed. Different types of arborist activities led to various types of physiological feedback, as shown by the analysis of variance. According to our results, there is a difference between physical workloads, associated with activities such as tree felling, tree climbing, or chainsaw maintenance, and cognitive workloads, such as supervision or observation, in relation to blood pressure. Blood pressure was higher for activities that involved a cognitive workload. Before and after any activity, handgrip strength was positively associated with hand size. After any activity, greater changes in handgrip strength of the participant's right hand were associated with needing more time to successfully complete a peg test, which represents a greater cognitive burden. Our results suggest that arborists deal with physical activities such as tree felling, tree climbing, working with a chainsaw, and mental activities (supervising or observing) which were identified as two different groups correlated with hand grip strength, blood pressure, manual dexterity, and spatial awareness. In conclusion, the tree-climbing activity appeared to be the least stressful, and psychological stress appeared to have a greater impact on the health of observers and supervisors in the study group. This can be applied to other professions in many fields, including industries where workers face both physical and cognitive workloads.

## 1 Introduction

The well-being of forestry workers has been a concern for many years. Ergonomic research among arboricultural professionals is a relatively new field of study. This study elaborates on new topics that could be important for understanding the work of arborists based on ergonomic concepts.

Arboriculture, a recently defined occupation, has been developed in cooperation with the forestry sector and has focused mainly on physical working conditions. From an ergonomic perspective, two major issues must be considered. First, people must adapt to heavy manual work, for which they use their own energy and simple tools. Second, human energy can be replaced by mechanized work, if possible. This means that the physical workload decreases, whereas the cognitive load increases [1]. Another aspect of arboriculture is that the working site is usually outdoors, where people can suffer from environmental factors, such as heat, cold, noise, vibrations and psychosocial stressors [[2], [3], [4], [5]].

Arboriculture work is manually and physically demanding. Moreover, arboriculture work also has a cognitive component, such as the responsibility of site safety management, especially for supervisors. The cardiovascular system responds to physical and cognitive stress by increasing heart rate and blood pressure. As Stramler [6] described, workload can be interpreted as the mental or physical effort that people expend when performing a specific task. Techniques used to assess workload can be either physiological or cognitive [7], which assess different responses. Subjective and objective work measurement techniques can be used to measure these two components. Feelings and perceptions can be measured by subjective measurement techniques, such as the NASA-TLX: Task Load Index, as mentioned by Hart and Staveland [8]; SWAT, subjective workload assessment techniques [9]; and SRTD, subjective rating of task difficulty [10]. Mital and Govindaraju [11] mentioned that subjective evaluations may be perceived as less reliable. According to Kopardekar [12], objective and quantitative techniques measure physiological components such as heart rate, blood pressure, muscle tension (strength), body temperature, aerobic capacity, electromyography, pupillary dilation, and speech analysis. Tycho et al. [1] introduced a rate-pressure product, given by the product of heart rate and systolic blood pressure, as an objective measure to distinguish between a cognitive workload task and a cognitive stress task for physical and cognitive workload.

Another risk factor related to arborists’ work includes falls from height, as mentioned by the National Institute of Occupational Safety and Health and Fatal Accident Circumstances and Epidemiology reports (NIOSH, 2000), the OSHA report on falls from scaffolds (OSHA, 1979), the OSHA report on falls from elevated platforms (OSHA, 1991), McCann (2003) for deaths in construction, and the study of HSE (2003) for falls from height in various industrial sectors [13]. Aneziris et al. [13] proposed a model that quantifies the probability of a fall from ladders, roofs, scaffolds, holes in the ground, moveable platforms and stationary vehicles, but a model predicting the probability of falling from a tree has not been developed. Although a complete picture of accidents involving arborists Europe is not available, Robb & Cocking [14] found that aerial chainsaw use was the most dangerous arborist activity in the United Kingdom. Training on rescue techniques, inspection, and work at height with equipment, seems to be neglected and is of particular importance since this neglect could lead to accidents with severe injuries, as reported by Jones [15], Statham and Roebuck [16], Longo et al. [17], Ferreira et al. [18], and Staněk et al. [19]. Neville [20] suggested tree-risk decision making as a process to ensure a confident and professional stance for sensible risk management policies and safe working conditions for arborists. As Bagagagiolo et al. [21] stated, small companies are less willing to invest in initiatives aimed at improving work safety. This should be considered when developing any successful initiative, as many arborist companies are known to be small to medium sized.

In general, arboricultural tasks include climbing trees, felling, cabling, pruning, removing trees, and other operations that require physical effort and muscular strength. Previous studies on these tasks include Biocca et al. [22], who investigated the biomechanical overload risks related to rope-based access (tree climbing) work and recommended more in-depth studies on arborist safety when dealing with adverse or stressful conditions. Another study investigated the technical and economic features of maintenance operations involving most arboricultural tasks [23]. Biocca et al. [24] found that although all companies involved experienced professional arborists, working time was influenced by operator skill and fitness. Arborists must work efficiently, which means that they must expend minimal energy to accomplish arboricultural tasks and perform them without laboured breathing or muscular pain. Martinic et al. [25], in their study of loggers, claimed that the optimum work capacity that can be sustained during the day is approximately 40% of maximal oxygen consumption. The occupational health and safety of arborists are highly influenced by posture and machinery use. Analysing the postural attitude of workers as they interact with their work elements and environment is essential for evaluating and preventing biomechanical overload risk [26]. Cremasco et al. [26] also highlighted that in relation to typical arborist activities, such as wood chipper operations, sound knowledge and practical implementation of ergonomic principles are important for reducing risks to the well-being of operators in the workplace. Initiatives that enhance automation in the arborist's workplace may be beneficial, for example, a shift towards the mechanisation of current aerial rigging activities. According to Aalmo et al. [27], improving one work task may negatively affect other work tasks, and automation should only be introduced to a worksite after considering all impacts on the entire system. The topic of tree climbers' health and safety has been the centre of attention of bodies such as the Awarding Body Association International (ABA), International Society of Arboriculture, Health and Safety Executive UK (HSE), Occupational Safety and Health Administration, and the U.S. Department of Labor (OSHA) [28]. Bridge [29] and Mazzocchi et al. [30] recommended risk assessment for tree climbing arborists. ABA offers professional training and evaluation, as was done during our study. One of the most common topics in arboricultural research is the costs and benefits of trees in cities. Roya, Byrne, and Pickering [31] summarized the social, economic, health, visual, and aesthetic benefits and ecosystem services, such as air quality, carbon, storm water, energy, habitat, noise, and microclimate-related ecosystem benefits. There are also negative issues, such as social hazards related to tree stability, insects, allergies, maintenance costs, pruning, and restoration of damage caused by tree failure or branch fall [31]. Ellison [32] quantified the risk of trees describing the impact on vehicles, pedestrians, and structures; however, the risk of tree failure while arborists are working is often neglected.

Research on arborist handsaws conducted by Mirka, Jin, and Hoyle [33] involved experiments on the efficiency of saw design and the height of sawing activity on the biomechanical response of the upper extremities. The authors used electromyography and subjective assessment to demonstrate the effects of saw design and work height. They stressed the importance of a neutral posture during work and appropriate sizing of the handle of the tool to reduce effort on the hand, wrist, and shoulder to minimise the development of carpal tunnel syndrome and hand/wrist tendonitis [33]. Other authors have focused on musculoskeletal disorders involving repetitive movements of the upper limbs [34,35]. Overall, little is known about the work of arborists in the context of ergonomic performance and how this relates to work safety. As reported by Ferreira et al. [18], working hours influence the physical and mental state of workers. The safety of work techniques, productivity, and level of fatigue are useful topics for research.

This study aimed to show the difference between the physical and cognitive activities of arborists combined with selected factors of workload, such as blood pressure, pulse, handgrip strength, other anthropometric measurements, and specific tests for manual dexterity and spatial awareness.

The following hypotheses were tested.

Hypothesis 1 Blood pressure is negatively correlated with handgrip strength after an activity.

Hypothesis 2 Different activities (supervising, tree felling, tree climbing, observing, and chainsaw maintenance) have different physiological feedback effects.

Hypothesis 3 There is a difference between physical (tree-felling, tree-climbing, and chainsaw maintenance) and cognitive workloads (supervising and observing) in relation to blood pressure.

Hypothesis 4 Handgrip strength is positively correlated with hand size before any activity.

Hypothesis 5 Handgrip strength is positively correlated with hand size after any activity.

## 2 Materials and methods

### 2.1 Research setting and participant selection

This study investigated the typical activities performed by arborists, including physical activities such as tree felling, tree climbing, or working with a chainsaw, followed by cognitive activities such as supervision and observation. The research was conducted in an outdoor setting at the Training Forest Enterprise Masaryk Forest Křtiny and indoors (classroom/workshop/ergonomics laboratory) at Mendel University in Brno. The participants included 10 volunteers aged between 17 and 48 years who were either attending or observing an ABA International arborist training course. Participants had at least three years of industry experience in arboriculture, with the exception of the youngest participant. The average height and weight of the participants were measured, along with body mass index (BMI), hand measurements, and spirometry. The ABA qualification guidelines ensured that the candidates undertook activities according to the same prescribed standards.

### 2.2 Research activities

The characteristics of the activities include the following:

**Activity A:** Supervising site safety while monitoring others’ activities. Supervision included surveillance of the activities to ensure that no harm occurred to the participants during their usual activities. The supervisor had to be ready to provide first aid if needed. They also ensured that the site risk assessment (mainly cognitive workload) was monitored, completed, and followed.

**Activity B:** Tree felling, felling, and crosscutting trees with a chainsaw and related tasks (mainly physical workload).

**Activity C:** Tree climbing, pruning branches, demonstrating aerial rescue methods, and related tasks. For activities B and C, the participants followed the criteria of the ICC/ECC1 (Chainsaw Maintenance & Crosscutting Techniques), ICC/ECC2 (Basic Tree Felling Techniques), and A1 (Tree Climbing, Hand Saw Use & Aerial Rescue Techniques) records of assessment. ABA qualification documents such as ICC/ECC1 [28] for chainsaw operations contain a complete breakdown of the tasks (mainly physical workload) undertaken (Table 1).

**Table 1:**
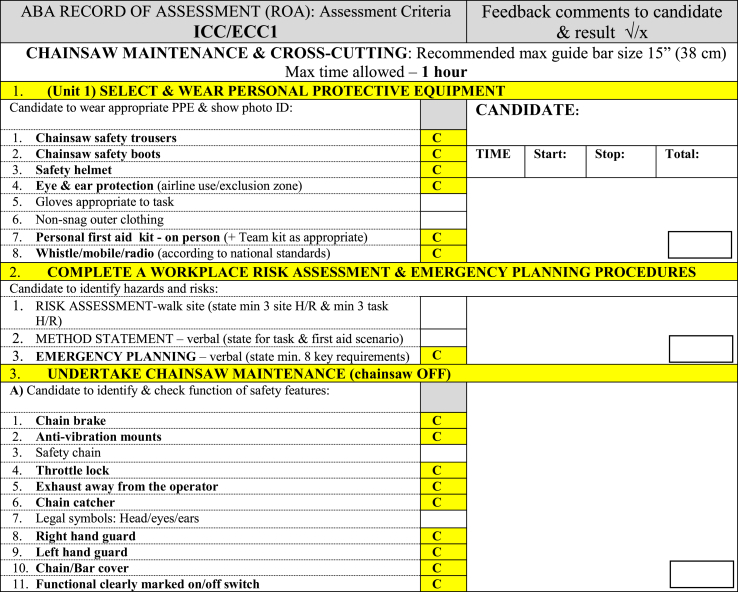
Chainsaw qualification safety standards.[28]

**Activity D**: Observing and monitoring the activities of others. During the activities, two participants undertook the role of observers to familiarise themselves with the ABA qualification assessment standards to understand what was involved and to provide support if needed (mainly cognitive activity). This type of role can be undertaken by ground staff at arboricultural worksites.

**Activity E:** Chainsaw maintenance workshop (a combination of physical and cognitive workload).

### 2.3 Data collection and outcome measures

For consistency, two researchers, with help from the same individuals each time (activity D observers), undertook the measurements with the dynamometer, Soew's cube, and peg test, as described below. These measurements were taken uniformly immediately before and after the activity. The researchers were trained before conducting the assessments.

#### 2.3.1 Strength measures

Handgrip strength is used to evaluate hand function, disease severity, and the effectiveness of treatment but can also measure general health, level of physical activity, and precision of overall strength [[36], [37], [38]]. As Mathiowetz et al. [39] stated, hand grip strength is the strength of the flexor muscles in the palm of the hand. The extensor muscles play a secondary role in grip strength, which assists the intrinsic muscles. Occupational therapists routinely measure grip strength in clinical settings for hand therapy or occupational rehabilitation [[40], [41], [42]]. According to Innes [43], grip strength can be evaluated to assess upper limb impairment or work capacity in people with hand injuries, evaluate people with disabilities, and measure treatment efficacy for people with disabilities. In our study, grip strength was evaluated to assess overall fitness [44] and determine the level of effort exerted [45,46].

Grip strength was measured using a dynamometer (DHD-1 digital hand dynamometer, SAEHAN, type SH1001, UK), which each person used in the same way, with the elbow tucked into the side and positioned at a right angle. The instrument was squeezed for 10 s in each case. The test was performed on both hands, typically with three squeezes per hand, and the average result was recorded.

#### 2.3.2 Cognitive measures-Spatial awareness

Cognitive ability was tested by analysing spatial awareness, which is the organised awareness of objects in the space around us, as well as an awareness of our body's position in that space. Understanding people's knowledge and feelings in the study of human spatial cognition has led researchers to examine the structure and processes of spatial knowledge from a cognitive, behavioral, or neurophysiological perspective, as mentioned for example by Golledge and Stimson [47] or Ishikawa [48]. People with poor spatial awareness tend to struggle with visual perception. For example, this can involve bumping into objects on a work site or standing too far from or too close to objects [49,50]. For arborists working at height within a tree crown, spatial awareness is critical; for example, arborists need to be aware of the distance between their tensioned rope systems or lifelines and the sharp edges of a handsaw. A lack of spatial awareness can quickly result in accidental cutting through lifelines, resulting in serious or fatal injuries. The participants' level of spatial awareness was evaluated using a cube test. The wooden cube, known as Soew's cube, consists of six differently shaped parts that had to be combined into a single cube shape. The time taken to complete the cube test was recorded. Soew's cube is used in psychological diagnostic practices [[51], [52], [53]]. Soew's cube is a manipulation test focused primarily on spatial imagination with use, for example, in professional counseling, as mentioned by Komárková and Vašina [54]. The author of the original method from 1953 is Bluemenfeld. The method is currently available at university workplaces, e.g. Palacky University Olomouc, Charles University and Mendel University in Brno (Czech Republic) [51].

#### 2.3.3 Cognitive measures-Finger dexterity

Cognitive ability was further evaluated using finger dexterity as monitored by the peg test, which evaluates fine motor skills, as confirmed by Okura and Yoon [55]. The peg test requires extensive use of the fingertips to place pins on a peg board. The peg test is timed; thus, speed and accuracy are considered in scoring. This manual dexterity test involves having the participant place round pegs in holes on a punched-out plastic board. Their ability to grab and place objects quickly is evaluated. This test has several different applications, including assessing limitations caused by carpal tunnel syndrome and dexterity problems caused by hand and wrist injuries [6,56]. Arborists who use chainsaws are more prone to these types of problems, particularly female arborists [56].

Cube and peg tests are often used to measure handgrip limitations [[57], [58], [59]]. in medical research, for example, for people after stroke [60], carpal tunnel syndrome [61], the aging population [[62], [63], [64]], rheumatic disease [65], and sensorimotor disability [49,66]. These measurements are uncommon in arboriculture studies.

#### 2.3.4 Blood pressure

The cardiovascular system responds to both physical and cognitive stress at work, which can affect emotional function. In recent years, the physical demand of jobs has decreased, and employees face increased cognitive workloads, including supervisory activities, as mentioned by Tycho et al. [1]. The cardiovascular system can be used to monitor stress response by monitoring increases in both heart rate and blood pressure. Blood pressure can be used to measure cognitive and physical workload [1].

Systolic (pressure 1) and diastolic (pressure 2) blood pressures were measured. Blood pressure indicates the force exerted by blood on the walls of the arteries. The first cardiac phase is pumping (systole), followed by rest (diastole) with regular rotation. First, the heart contracts the left ventricle and pumps blood into the bloodstream (systolic and upper blood pressure). In the second phase, the heart rests and the blood does not pump, representing diastolic pressure and the resistance of the vascular bed [6]. The difference in diastolic pressure was used to determine the correlation between blood pressure and handgrip strength (Hypothesis 1). Pulse rates (heart rates) were measured using an OMRON M6 device.

### 2.4 Data analysis

Correlation analysis was used to assess the association between physically demanding arborist activities and subsequent handgrip strength. To analyse physical workload, the blood pressures and handgrip strengths of arborists were measured before and after the activities and the difference was recorded. The results are presented in correlation tables and correlation coefficients were deemed significant if p < 0.05. The correlation coefficient, which expresses the strength of the association, was determined.

In this study, we did not compare the exact pressures among the participants; instead, we only compared the differences between values measured before and after activities. This eliminates the natural differences in pressure levels between people, which can have several origins.

One-way analysis of variance was used to assess the differences in physical workload indicators (differences in blood pressure and handgrip strength) depending on activity (A–E). The F test was performed for the physical workload factors. Null hypotheses were rejected if p < 0.05. The mean changes in handgrip strength and blood pressure, with corresponding 95% confidence intervals, for each activity are graphically displayed for clarity and better interpretability.

Using correlation analysis, the association between handgrip strength of the right and left hands and anthropometric factors was also assessed. Significant correlations (p < 0.05) are highlighted. Correlation coefficients (absolute value) ranged from 0 to 1, where 0.00–0.20 is very low association, 0.20–0.40 is low, 0.40–0.60 is moderate, 0.60–0.80 is high moderate, and 0.80–1.00 is a very high strength association. All analyses were performed using IBM SPSS Statistics software.

### 2.5 Ethics approval and consent

This study was reviewed and approved by the Ethics Committee of Mendel University in Brno (approval number: 25/2022). All the participants provided informed consent to participate in the study. All the participants provided informed consent for the publication of anonymised case details and images. Parental consent was obtained for participation in the study and publication of anonymised case details and images for those aged under 18 years.

## 3 Results and discussion

The well-being at work of forestry workers has been a topic which has been studied over a longer period of time than professionals working in arboriculture [3,5,[25], [26], [27]]. Ergonomic research in arboriculture is a relatively new field of study and the aim of this paper is to advance both knowledge and an understanding of the physical and cognitive aspects of the working environment. The most important work factor, mentioned by numerous authors, is safety at work [14,16,17,19]. As arborists work in a high-risk environment, the combination of grip strength and resistance to psychological stress are also very important attributes in maintaining a safe workplace. Many authors confirm that arboriculture is demanding due to the workplace being outdoors and environmental effects [5], such as exposure to extreme temperature, noise or vibrations, but also psychosocial/cognitive aspects, including time schedule issues [2,18], individual characteristics [3,24] or levels of workers stress [4,22]. Another aspect is that people working in arboriculture are often engaged in heavy manual work which requires human energy [4] and basic tools which [33] although to some extent can be replaced by mechanized work, this still does require cognitive demand [1].

As shown in Table 2, the 10 participants in our sample were all men, aged 17–48 years, with an average age of 33 years, which is at the lower end of the average employment age scale. As stated by da Silva et al. [67], the proportion of workers aged 50 years and over, employed in the forestry sector in Europe increased between 2005 and 2015. Simultaneously, the number of workers aged 35 years and younger has continuously decreased [67]. In this study, the average height and weight of the participants were 179 cm and 86 kg, respectively.

**Table 2:** Descriptive statistics of participants.

	N	Minimum	Maximum	Mean	Standard deviation
Size_of_hand_cm	7	18.00	21.00	19.50	1.08
Age	10	17.00	48.00	33.40	11.19
Height_cm	10	170.00	187.00	179.00	6.25
Weight_kg	10	70.00	110.00	85.90	12.89
Body mass index	10	21.04	32.85	26.82	3.82
Wooden_cube_min	9	0.34	7.34	2.72	2.36
Finger_test_LEFT_hand_min	9	2.37	3.44	2.63	0.41
Finger_test_RIGHT_hand_min	9	2.20	3.05	2.50	0.33
Finger_test_BOTH_hand_min	9	1.31	2.35	1.57	0.39
Spirometer_litre	9	3.20	5.20	4.31	0.60

### 3.1 Relationship between grip strength and blood pressure after any activity

Workload assessment techniques include physiological and cognitive [7] measures, which differ according to the type of response. The use of subjective [[8], [9], [10], [11],^33^] or objective [12,[33], [34], [35]] work measurement techniques was confirmed by various studies. Our paper focuses on objective, quantitative, work measurements techniques, as mentioned by Kopardekar [12], which are based on physiological measures such as heart rate, blood pressure, muscle tension or grip strength.

Hypothesis 1 There is a negative correlation between blood pressure and handgrip strength after a performed activity.

Table 3 highlights associations between changes in handgrip strength and blood pressure for all activities (A–E) and not for each separate activity. The focus was on the correlation between changes in blood pressure and handgrip strength before and after an activity. We found that the correlation between changes in diastolic blood pressure and grip strength of the left hand was significant. For all activities (A–E), increased blood pressure after an activity was associated with decreased handgrip strength. This correlation was stronger for the left hand and weaker for the right hand.

**Table 3:** Correlation between blood pressure and hand grip strength for all activities.

		Pressure_2_DIFFERENCE
Left_hand_DIFFERENCE	Pearson correlation	−0.427*
	Significance (p value)	0.030
	N	26
Right_hand_DIFFERENCE	Pearson correlation	−0.298
	Significance (p value)	0.116
	N	29

Conclusion: Greater differences in blood pressure (diastolic, before and after all activities) were associated with lower hand grip strength, in the left hand (Table 3). Thus, Hypothesis 1 was confirmed. An assessment of handgrip strength can be used for the evaluation of hand function, measurement of general health and a determination of overall strength, as confirmed by various studies [[36], [37], [38], [39],^43^,^44^]. For the purpose of our study, grip strength evaluation is part of an overall fitness assessment [44] and determination of the level of effort exerted [45,46]. The results from Table 3 and the following Table 4 show that the cardiovascular system brings responses to physical stress via changes in heart rate and blood pressure, as summarized by Stramler [6].

**Table 4:** Analysis of variance showing the differences in physiological feedback between activities.

		Sum of Squares	freedom	F	Significance (p value)
Systolic blood pressure _AFTER	Between Activities	5009.865	4	4.835	**0.005**
	Within Activities	6217.583	24		
	Total	11227.448	28		
Systolic blood pressure _DIFFERENCE	Between Activities	912.562	4	0.612	0.658
	Within Activities	8941.300	24		
	Total	9853.862	28		
Diastolic blood pressure _AFTER	Between Activities	3090.978	4	3.701	**0.017**
	Within Activities	5011.022	24		
	Total	8102.000	28		
Diastolic blood pressure _DIFFERENCE	Between Activities	2045.006	4	3.169	**0.032**
	Within Activities	3871.683	24		
	Total	5916.690	28		
Pulse_after	Between Activities	1370.278	4	2.894	0.065
	Within Activities	1538.667	13		
	Total	2908.944	17		
Pulse_difference	Between Activities	1856.611	4	2.036	0.148
	Within Activities	2963.000	13		
	Total	4819.611	17		
Left_hand_AFTER	Between Activities	294.485	4	1.037	0.412
	Within Activities	1491.229	21		
	Total	1785.714	25		
Left_hand_DIFFERENCE	Between Activities	97.115	4	0.327	0.857
	Within Activities	1559.354	21		
	Total	1656.468	25		
Right_hand_AFTER	Between Activities	991.553	4	3.231	**0.030**
	Within Activities	1841.476	24		
	Total	2833.029	28		
Right_hand_DIFFERENCE	Between Activities	178.264	4	1.387	0.268
	Within Activities	771.105	24		
	Total	949.369	28		

### 3.2 Differences in physiological feedback between activities: analysis of variance

Hypothesis 2 Different activities (A–E) have various physiological feedback effects.

Table 4 shows systolic and diastolic blood pressure, heart rate, and grip strengths for both hands measured after an activity and differences between pre- and post-activity measurements. The analysis of variance showed significant between-activity differences for the indicators, systolic and diastolic blood pressure after an activity, diastolic blood pressure change, and grip strength of the right hand after an activity.

assumptions of normality and homogeneity of variances were tested using the Shapiro–Wilk and Levene's tests. These assumptions were accepted at the 0.05 level. At the 5% level of significance, the mentioned differences were proven, hypothesis 2 is confirmed.

### 3.3 Cognitive and physical workload and blood pressure after an activity

Hypothesis 3 There is a difference between physical (tree felling, tree climbing, chainsaw maintenance) and cognitive workload (supervising, observing) in relation to blood pressure.

**Fig. 1:**
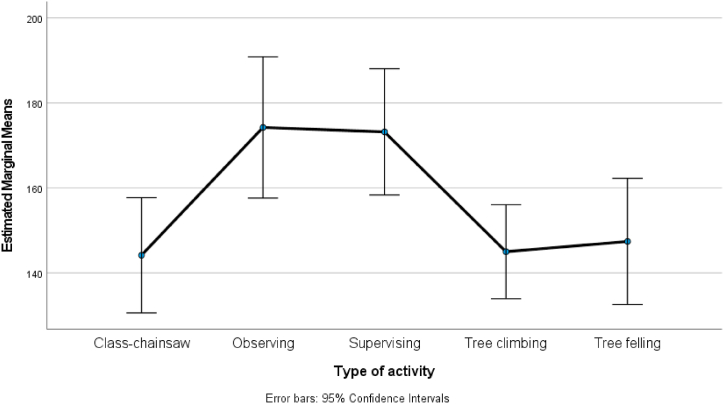
Systolic pressure after activities A-supervising, B-tree felling, C-tree climbing, D-observing, and E-chainsaw maintenance workshop (p-value of F-test 0.005).

**Fig. 2:**
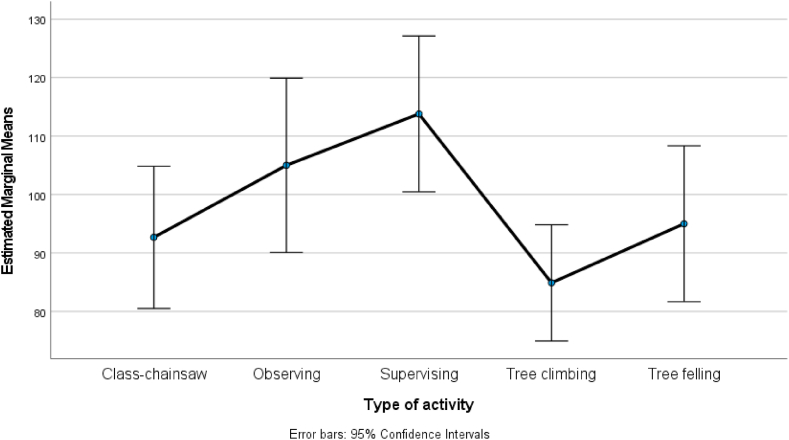
Diastolic pressure after activities A-supervising, B-tree felling, C-tree climbing, D-observing, and E-chainsaw maintenance workshop (p-value of F-test 0.017).

Fig. 1, Fig. 2 highlight differences at the 5% level of significance for the means of various indicators related to different activity types (A–E).

Fig. 1 shows the differences in mean systolic blood pressure after each of the following activities: A-supervising, B-tree felling, C-tree climbing, D-observing, and E-chainsaw maintenance workshop.

The largest difference in systolic blood pressure was observed between cognitive activities (A-supervising and D-observing) and physical activities (B-tree felling, C-tree climbing, and E-chainsaw workshop). By using post-hoc tests the mean systolic blood pressure values after activities A and D were significantly higher than those after activities B, C, and E.

Interpretation: Our results suggest that blood pressure was higher after cognitive activities, which might be related to emotional responsibility for others and the possibility of harm due to potential risks or stress.

Interestingly, Fig. 2 confirms that diastolic blood pressure was also significantly higher after activities A and D than activities B, C, and E.

Hypothesis 3 was confirmed, there is a difference between physical and cognitive workload in relation to blood pressure.

### 3.4 Association between handgrip strength and hand size before any activity

Hypothesis 4 There is a positive correlation between handgrip strength and size of the hand before any activity.

Hand size (palm, from the wrist to the end of the middle finger) of the right and left hands was significantly associated (p < 0.05) with handgrip strength before an activity (Table 5). This positive correlation suggests that as hand size increases, so does handgrip strength before the activity.

**Table 5:** Correlation between handgrip strength and anthropometric measurements before any activity.

	Left_hand_BEFORE			Right_hand_BEFORE		
	Pearson correlation	Significance (p value)	N	Pearson correlation	Significance (p value)	N
Size_of_Hand_cm	**0.476**	**0.046**	18	**0.515**	**0.017**	21
Age	0.114	0.596	24	−0.115	0.567	27
Height_cm	0.185	0.387	24	**0.486**	**0.010**	27
Weight_kg	−0.059	0.784	24	−0.117	0.562	27
Body mass index	−0.15	0.484	24	**−0.377**	**0.053**	27
Wooden_cube_min	0.177	0.43	22	**0.365**	**0.073**	25
Finger_test_LEFT_hand_min	**−0.401**	**0.064**	22	**−0.74**	**0.000**	25
Finger_test_RIGHT_hand_min	−0.374	0.086	22	**−0.659**	**0.000**	25
Finger_test_BOTH_hand_min	−0.297	0.179	22	**−0.344**	**0.092**	25
Spirometer_litre	0.168	0.454	22	0.313	0.128	25

In contrast, handgrip strength before an activity was negatively associated with peg test scores. Time taken to successfully complete the peg test increased as handgrip strength measured before the activity decreased. Greater handgrip strength was associated with less time needed to successfully complete the test. This suggests that people with less handgrip strength may require more time for the peg test, as they might have lower finger dexterity.

For spatial awareness, measured using Soew's cube, greater handgrip strength was associated with a longer time taken to complete the cube. People with lower handgrip strength needed less time to complete this task, suggesting that they had better spatial awareness. As highlighted earlier, difficulties with spatial awareness may be identified using the cube test [48], whereas difficulties with finger dexterity can be detected using the peg test [55]. For example, finger dexterity is important for arborists when manipulating karabiner gate mechanisms and handling ropes and knots.

### 3.5 Association between handgrip strength and anthropometric measurements AFTER any activity

Hypothesis 5 There is a positive correlation between handgrip strength and size of the hand after any activity.

Changes in handgrip strength after any activity were only significant for the association between changes in right-hand strength and longer times taken to complete peg tests (Table 6). The peg test was performed with the left and right hands together (finger dexterity). We observed that greater changes in handgrip strength in the right hand after an activity, resulted in the participant needing more time to complete the successful peg test, which is a greater cognitive burden.

**Table 6:** Correlation between handgrip strength and anthropometric measurements after any activity.

	Left_hand_DIFFERENCE			Right_hand_DIFFERENCE		
	Pearson correlation	Significance (p value)	N	Pearson correlation	Significance (p value)	N
Size_of_hand_cm	0.125	0.623	18	0.111	0.632	21
Age	0.098	0.648	24	0.257	0.196	27
Height_cm	0.329	0.116	24	0.085	0.673	27
Weight_kg	0.209	0.328	24	**0.355**	**0.069**	27
Body mass index	0.052	0.809	24	**0.342**	**0.081**	27
Wooden_cube	−0.141	0.531	22	−0.037	0.86	25
Finger_test_LEFT_hand	0.079	0.727	22	**0.446**	**0.026**	25
Finger_test_RIGHT_hand	0.132	0.557	22	**0.452**	**0.023**	25
Finger_test_BOTH_hand	0.352	0.108	22	**0.371**	**0.068**	25
Spirometer_litre	0.455	0.033	22	0.043	0.838	25

The aim of this study was to show the difference between physical and cognitive activities of arborists in relation to selected workload factors. The observed activities included tasks that were primarily physical (tree felling, tree climbing), then cognitive (supervision, observation) and a combination of both (chainsaw maintenance). Consistent with the confirmation of Hypothesis 1, all observed activities showed that increased blood pressure after an activity was associated with decreased hand grip strength, particularly of the left hand. This finding confirms the opinion of many authors that work in arboriculture still requires physical fitness [3,5,25] and that physiological measures such as blood pressure and grip strength may differ depending on the activity performed, as Koperdekar [12] states. Hypothesis 2 confirms that different activities have different physiological effects, particularly for post-activity blood pressure and post-activity grip strength, especially for the right hand. It can be seen that not only grip strength but also palm size (Hypothesis 4) can be useful indicators for monitoring the health status of the arborist or for professional advice. Similarly, the use of dexterity tests or spatial dexterity tests (Hypothesis 5) can usefully complement the information on an arborist's skills, as has been mentioned for other occupations, for example by Komárková and Vašina [54] and others [[51], [52], [53]].

Hypothesis 3 clearly confirmed the difference between cognitive activities - observation and supervising activities - and other, mainly physical activities. This result is consistent with Hypothesis 1 that, although arboriculture is primarily a physically demanding field, the proportion of physical work is decreasing (for example thanks to mechanisation) and space is being created for increasing activities of a cognitive nature. These include supervision, observation, control, decision-making processes in site safety management [15,16,20,28] or work under stressful conditions [22]. It is also evident that the work of an arborist is influenced not only by environmental factors but also by psychosocial stressors [[2], [3], [4], [5]]. This result also confirms the earlier findings of Stramler [6] or Biocca et al. [22], that the work of an arborist actually has not only a physical but also a cognitive component.

## 4 Conclusion

Arborists work in high-risk environments, especially when climbing trees, and a combination of good grip strength and resistance to psychological stress are important attributes for maintaining a safe workplace. Our results suggest that arborists deal with physical activities such as tree felling, tree climbing, or working with a chainsaw and with mental activities (supervising or observing), which were identified as two different groups correlated with hand grip strength, blood pressure, manual dexterity, and spatial awareness.

After any activity, a change in grip strength of the right hand (mostly used by arborists) was associated with manual dexterity. We observed that participants with larger hands had stronger grip strength. Stronger grip strength was associated with greater manual dexterity and less spatial awareness.

Overall, even in a test environment (during ABA skills assessments), the tree-climbing activity appeared to be the least stressful, which warrants further research to include complete rigging/dismantling operations. However, although not in a test environment, the effect of psychological stress (due to emotional responsibility for others) appeared to have a greater impact on the blood pressure of observers and supervisors within the study group. This indicates that although other arborist activities are more physically demanding, the mental burden is lower, as they have more control over their immediate environment, unlike observers and supervisors.

### 4.1 Limitations

This study was carried out with a limited sample of participants (10) and does not, as highlighted previously, represent the average age of the operators working as arborists in the forestry industry or include female participants. Therefore, further research with a larger, demographically diverse population sample is recommended to increase the validity and reliability of results. The authors are aware of the possible limitations of this study, including the use of different technologies for physiological and cognitive research. Objective research techniques [7] were used in the present study, which according to Koperdekar [12] are considered more reliable than subjective techniques [11]. To continue the research, it would be possible to use subjective measurement techniques, e.g. the Task Load Index and take inspiration from the work of Hart and Staveland [8] or subjective workload assessment techniques [9] or subjective task difficulty assessment [10]. It is also worth considering the use of the rate-pressure product developed by Tycho er al [1]. or a combination of electromyography and subjective assessment, as explained by Mirka, Jin and Hoyle [33].

## Funding

This study did not receive any specific grants from funding agencies in the public, commercial, or nonprofit sectors. Publication of the study was supported by 10.13039/501100008775Mendel University in Brno.

## Data availability statement

The data are not publicly available because they contain information that can compromise the privacy of the research participants. The data is available on request.

## CRediT authorship contribution statement

**Eva Abramuszkinová Pavlíková:** Writing – review & editing, Writing – original draft, Validation, Supervision, Methodology, Investigation, Formal analysis, Data curation, Conceptualization. **William Robb:** Writing – review & editing, Writing – original draft, Methodology, Formal analysis, Conceptualization. **Jakub Šácha:** Methodology, Investigation, Formal analysis, Data curation.

## Declaration of competing interest

The authors declare that they have no known competing financial interests or personal relationships that could have appeared to influence the work reported in this paper.
